# A Retrospective Cohort Study of Lung Cancer Incidences and Epidemiological Analysis in Saudi Arabian Population from 2006–2016

**DOI:** 10.3390/ijerph182211827

**Published:** 2021-11-11

**Authors:** Ahmad Almatroudi

**Affiliations:** Department of Medical Laboratories, College of Applied Medical Sciences, Qassim University, Buraydah 51452, Saudi Arabia; aamtrody@qu.edu.sa

**Keywords:** lung cancer, Saudi cancer registry, ASIR, CIR, epidemiological analysis

## Abstract

Introduction: Lung cancer is one of the leading causes of cancer-related deaths worldwide, including in Saudi Arabia. In the past few decades, the incidence and mortality has increased considerably among the Saudi population. The incidence of lung cancer has increased more than 3% in less than two decades. The current study focuses on analyzing the frequency of diagnosed lung cancer cases, age-standardized incidence rate (ASIR), and crude incidence rate (CIR) classified by year of diagnosis, age group, and different administrative regions of Saudi Arabia, based on the Saudi cancer registry (SCR) for the period of 10 years from 2006–2016. Methodology: The present study is a cohort study that focuses on the epidemiological analysis of lung cancer cases. The statistical analysis was performed by *t*-test, sex ratio, Kruskal–Wallis, and descriptive statistics through SPSS version 20.0. Results: A total of 4530 lung cancer cases were reported from January 2006 to December 2016. Among males, the ASIR range was reported as 1.2 to 12.3 per 100,000 cases in different regions of Saudi Arabia. Moreover, among females, the ASIR range was reported from 0.2 to 3.1 per 100,000 cases in different regions of Saudi Arabia. Additionally, the overall age-standardized incidence sex ratio was 3.2 per 100,000 cases. The incidence of lung cancer increased with age as 23% was reported in the age group of more than 75 years. Conclusions: The study concludes a rise in crude incidence rates and ASIR for lung cancer among Saudi population. Among males, the Eastern region had the highest overall ASIR followed by Tabuk region, and, among females, the highest overall ASIR was reported in the Eastern region followed by Riyadh and the Northern region.

## 1. Introduction

Cancer is one of the leading causes of death globally. WHO estimates highlights cancer is amongst the first three causes of deaths in more than 150 countries worldwide [1]. In 2020 alone, 19.3 million new cancer cases and 9.9 million cancer-related deaths were reported globally. The burden of cancer incidences, its prevalence, and mortality are growing rapidly, which is reflected in the population growth and aging worldwide. Along with these prominent risk factors, several others can be associated with lifestyle and regional socio-economic development. Lung cancer is a highly invasive, rapidly metastasizing, and prevalent cancer and is considered a prominent cause of cancer-related mortality among men and women globally. Though female breast cancer has surpassed lung cancer as the most commonly diagnosed cancer, with an estimated 2.3 million new cases (11.7%) in 2020, lung cancer is the second most common cancer, with 11.4% of the cases, and it remains the most common among males globally [1]. It is categorized into non-small cell lung cancer (NSCLC) and small cell lung cancer (SCLC) [2]. NSCLC is the most common form of lung cancer primarily caused by smoking and the use of tobacco products and is further categorized into adenocarcinoma and squamous cell carcinoma [1]. The lung cancer five-year survival rate is 4–17%, and around 80% of lung cancer patients are NSCLC [3]. Currently, primary procedures for early-stage lung cancer are surgical treatments [4], and late diagnosis reduces the chances of surgical treatment and diminishes long-term survival with increased morbidity towards the later stages. Awareness of lung-cancer-associated risk factors is the important step for efficient control, thereby decreasing the overall lung cancer mortality [5]. However, the effects of these risk factors differ regionally and spatially [6]. Geographical distribution and incidence of the disease can vary with lifestyle habits, cultural beliefs, sex and occupation, socio-economic development, availability of medical facilities, and accessibility/affordability to treatment services [5]. Socio-economic development may also be associated with several risk factors of lung cancer, particularly in developing countries of the world, such as exposure to usage of tobacco products and tobacco smoking, particularly in the younger population age groups [7]. In the Middle East and North Africa region, more than 46% of people are engaged in tobacco consumption practices, which is one of the leading causes of lung cancer worldwide. Although Western countries have been able to reduce smoking induced lung cancer over the years, the problem in developing countries is increasing each day due to prevalent socio-economic conditions. Occupation-associated risk factors are other oncogenic agents for lung cancer such as exposure to asbestos, radon, and other hazardous chemical exposure [8]. A study in Iran demonstrated that the main risk factor for lung cancer was smoking, followed by occupational irritants [6].

Globally, soaring cancer cases and increasing cancer burden has engulfed countries worldwide with increased incidences particularly in developing countries, and Saudi Arabia is no exception to this. The remarkable socio-economic development of the region, particularly in the last 40 years, has brought massive changes in the socio-economic structure sedative lifestyles, processed food consumption, and other behaviors. The socio-economic-induced lifestyle modifications are believed to be responsible for prevalence of many diseases including cancer in many developing countries [9]. In 1990, the total percentage of deaths due to cancer was approximately 5%, and the number increased and reached 12% in 2016 [10]. The incidences of lung cancer alone have increased 3.5-fold in the past two decades from 350 cases in 1990 to 1200 up to 2016.

The socio-economic development and improvement in medical facilities has also encouraged the government to establish plans for improving the medical care of its citizens, under which several initiatives have been taken. Annual collection, maintenance, and publication of cancer-related data and information is one of them. The current study aims to examine the epidemiological parameters of crude incidence rates (CIR) and age-standardized incidence rates (ASIR) of lung cancer classified by age group, year of diagnosis, and different administrative regions of Saudi Arabia for investigation and discussing the regional distribution of lung cancer incidences. A detailed observational epidemiological study has been performed to identify the distribution of lung cancer cases documented between 2006 and 2016 based on the Saudi cancer registry (SCR). Since more than half of Saudi Arabia’s population is less than 29 years old, significant initiatives are dedicated to revealing the incidence and mortality of cancer in society in order to implement a management, screening, and prevention strategy in the country. The study would aid in generating lung-cancer-related epidemiological information about the Saudi Arabian population and would ultimately help in understanding cancer trends, which could help in efforts to manage the incidence and mortality of cancer in the future.

The increases in the incidence of different types of cancer in the past decade could be due to the revolutionary change in socioeconomic status that has occurred in Saudi Arabia; therefore, a national plan should be established for cancer prevention, screening, and therapy. Concerning mortality, the decrease in its percentage among elderly people could be due to biological factors that should be investigated in the future.

## 2. Material and Methods

A retrospective observational population-based epidemiological study on lung cancer based on cancer registry data by the Ministry of Health, Saudi Arabia, has been attempted [10]. The cohort study is based upon the published data of Saudi Arabia cancer registry from January 2006 to December 2016. Since the data have been obtained from the open access public domain, available through the SCR annual report, the ethical clearance was not required for the same [10]. SCR presented a comprehensive report from January 2006 and December 2016 for 13 administrative regions, incidence with the percentage of cases, ASIR and CIR stratified by year of diagnoses, sex, and administrative regions. The current study included only Saudis (citizens of Saudi Arabia), and non-Saudi residents were excluded [10]. We used SPSS version 20.0 (IBM Corporation, Armonk, NY, USA) for statistical analysis. In different regions of Saudi Arabia, ASIR and CIR from the SCR reports were recorded for the year 2006 to 2016, and the difference between them was calculated to identify the lung cancer trend amongst men and women. An independent samples t-test was performed for comparing ASIR and CIR incidences of lung cancer among females and males. A nonparametric Kruskal–Wallis H-test was also conducted for correlating ASIR and CIR between different administrative regions of Saudi Arabia. Additionally, the male/female ratio of lung cancer was also calculated from the age-specific incidence rate (AIR), ASIR and CIR categorized by year of diagnosis, age groups, and administrative regions. The overall percentage of stage distribution of lung cancer from 2006 to 2016 was also identified among males and females. Stages of cancer from SCR were categorized into regional, distant, localized, and unknown stages [11]. A five-year class interval has been set to categorize the population data into 16 standardized age groups: 0–4, 5–9, 10–14, 15–19, 20–24, 25–29, 30–34, 35–39, 40–44, 45–49, 50–54, 55–59, 60–64, 65, 69, 70–74, and 75 and above years for the study.

## 3. Incidences of Lung Cancer among Males

**Figure 1:**
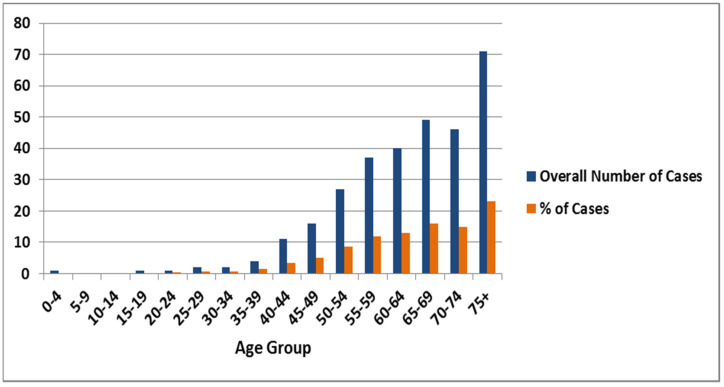
Graphical representation of overall number and percentage of lung cancer cases in males during 2006 to 2016.

Based on the SCR data, a total of 3388 lung cancer cases were reported in the period of 10 years between January 2006 and December 2016. From 2006 to 2016, incidence of the number of lung cancer cases increased steadily, which was only 232 in 2006 (6.8%) and increased to 297 in 2016 (8.8%). From 2006 to 2016, the overall number and percentage of lung cancer was 308 cases (9.1%) per year in males. Males in the >75 years age group were observed to be most frequently affected by lung cancer (71 overall cases per year, 23%), followed by the age group of 65–69 years (49 overall cases per year, 15.9%) and 70–74 years with 15% of cases. The lowest percentage of cases was observed in the young age group (0–39 years), ranging between 0.00–0.08% amongst 0–39 age groups. (Figure 1). The overall percentage of lung cancer incidences among the male population has fluctuated over the 10 year time period; it was 6.8% in 2006, steadily increased to 10.1% in 2011, then declined to 9.9% and 9.6% in 2012 and 2013, then increased to 10.4%, and further declined to 8.8% in 2016 Table 1.

## 4. Incidences of Lung Cancer among Females

**Figure 2:**
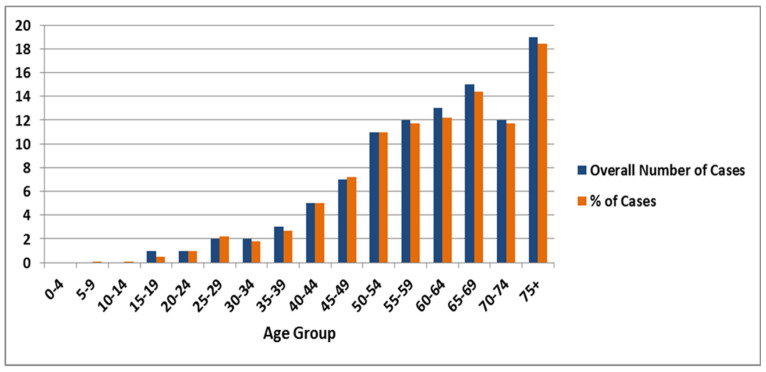
Graphical representation of number and percentage of lung cancer in females between 2006 and 2016.

Based on the SCR data, a total of 1142 lung cancer cases were reported in the period of 10 years between January 2006 and December 2016 among females in the Saudi population. From 2006 to 2016, the incidence of the number of lung cancer cases increased drastically, which was only 80 in 2006 (7%) and increased to 148 in 2016 (13%). From 2006 to 2016, the average number and percentage of lung cancer cases were 104 (9.1%) per year in females. Similar to males, the female population in the >75 years age group was observed to be most frequently affected by lung cancer (18.4% of overall cases) followed by the age group of 65–69 years (14.4%) and 60–64 with 12.2% of overall cases in all the 16 age groups. The lowest percentage of cases was observed in the young age group (0–24 years), ranging between 0.00–0.08% amongst 0–24 age groups (Figure 2). The overall percentage of lung cancer incidences amongst female population has also fluctuated over the given 10 year time period from 2006–2016; it was 7% in 2006, steadily increased to 11.1% in 2011, decreased to 8% in 2015, and then increased drastically to 13% in 2016 (see Table 1).

## 5. Lung Cancer: ASIR and CIR Incidences

**Figure 3:**
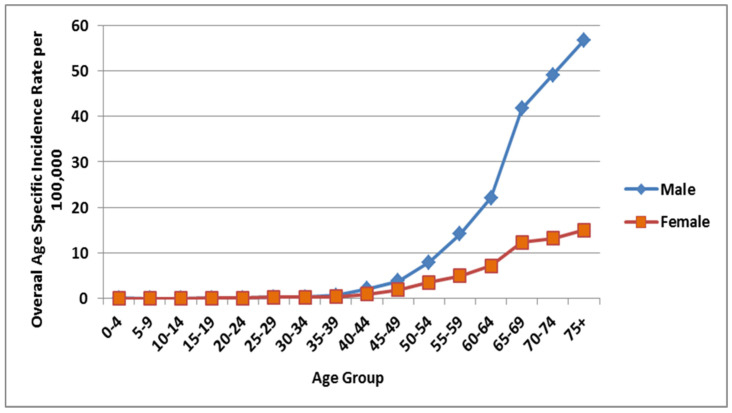
Overall age specific incidence rate of lung cancer cases from 2006 to 2016 per 100,000 among male and female.

The highest overall age specific incidence rate (AIR) of lung cancer cases from 2006 to 2016 was observed among the males falling in the in the age group of >75 years and over (56.7%), followed by 70–74 (49.1%) and 65–69 (41.7%) per 100,000 males (Figure 3). Similarly, amongst the females, the highest AIR was also observed for the age group >75 with a 13.2% rate, and then it declines gently with reducing age. A positive correlation has been observed between the increasing age and number of lung cancer incidences among males and females in the region, i.e., the number of lung cancer incidences can be seen increasing with age; therefore, most of the lung cancer incidences can be attributed to age/age-related factors in the region. Although both male and female incidences have increased over the years for lung cancer, the increase has been sudden and drastic for the male population as compared to the female population in the region. (Figure 3).

**Figure 4:**
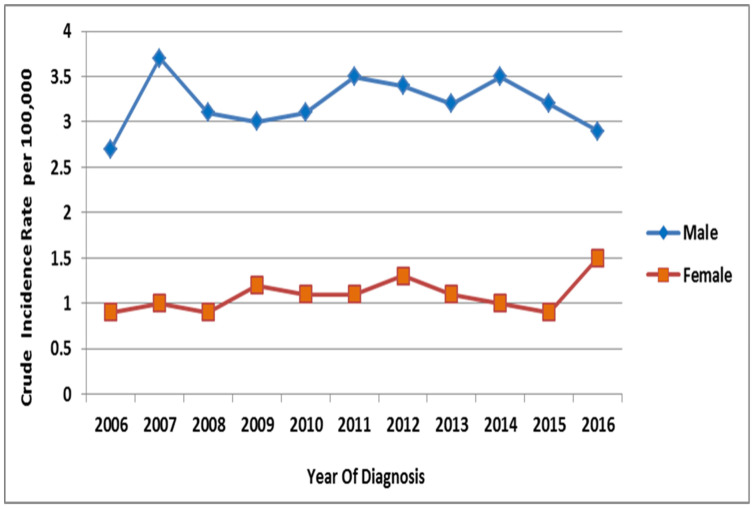
Crude incidence rate (CIR) of lung cancer per 100,000 among males and females during 2006 to 2016.

The CIRs of lung cancer cases in Saudi males upon stratified year-based analysis from 2006 to 2016 per 100,000 males reflect a dynamic picture, where they were high during the initial years in 2006, 2007, and 2008 and ranged between 3 to 3–4, then decreased from 2007 to 2008, remained stable until 2010, then increased in 2011 and remained stable until 2015, and then drastically decreased from 3.6 in 2015 to 1.9 per 100,000 in 2016 (Figure 4). In 2006, a CIR of 2.7 per 100,000 males was estimated. A CIR of 3.7 per 100,000 males was the highest rate documented in 2007. From 2006 to 2016, the overall CIR per 100,000 males was 3.2 (95% CI, 3–3.4). The two independent sample *t*-tests were also applied to CIR among males and females and found that Saudi males had significantly higher CIR compared to Saudi females (t(20) is 20.128, *p* < 0.0001). The overall crude incidence male/female ratio per 100,000 males from 2006 to 2016 was 3 (Figure 4).

The CIRs of lung cancer cases per 100,000 females in Saudi females, upon categorization by the year of diagnosis from 2006 to 2016, remain stable from 2006 to 2008, then increase slightly in 2009, remain stable until 2015, and then increase in 2016 (Figure 3). A CIR of 0.9 per 100,000 females was estimated in the year 2006. A CIR of 1.5 per 100,000 females was the highest rate reported in the year 2016. The overall CIR from 2006 to 2016 was 1.1 per 100,000 females (95% CI, 1–1.2).

## 6. CIR by Geographical Regions

**Figure 5:**
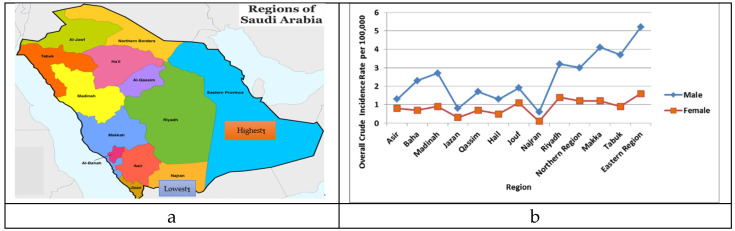
(**a**). Lung cancer and its incidence in different regions of Saudi Arabia: CIR by Geographical regions in Saudi Arabia. Source: https://www.geocurrents.info/cartography/customizable-maps-iran-saudi-arabia-malaysia-indonesia/attachment/regions-of-saudi-arabia-map. (**b**): Overall CIR of lung cancer cases per 100,000 among males and females according to administrative regions during 2006 to 2016.

In addition, the overall CIR of lung cancer stratified by the administrative regions of Saudi Arabia per 100,000 males and females was also computed. The highest overall CIR for males was observed in the Eastern region at 5.2, followed by the Makka region at 4.1 per 100,000 females. For females, the highest was also observed in the Eastern region, i.e., 1.6. It was observed that the result of the Kruskal–Wallis test was statistically significant for these regions in comparison to other areas of Saudi Arabia (*p* < 0.001). The Najran and Jazan region reported the lowest overall CIRs at 0.6 and 0.8, respectively. The highest crude incidence sex ratio was documented by Najran at 6, followed by Tabuk at 4.1 (Figure 5a,b).

The overall CIR of lung cancer was also calculated upon stratification by the different regions of Saudi Arabia per 100,000 females (Figure 4). The Eastern region had the highest overall CIR for lung cancer at 1.6 per 100,000 females, followed by the Riyadh region at 1.4 per 100,000 females. It was observed that the result of the Kruskal–Wallis test was statistically significant for these regions in comparison to other areas of Saudi Arabia (*p* < 0.001). The Najran region documented the lowest overall CIR at 0.1, followed by Jazan at 0.3 per 100,000 females. Regionally, the highest CIR was recorded for the Eastern region and the lowest for the Najran region (Figure 5a,b).

**Figure 6:**
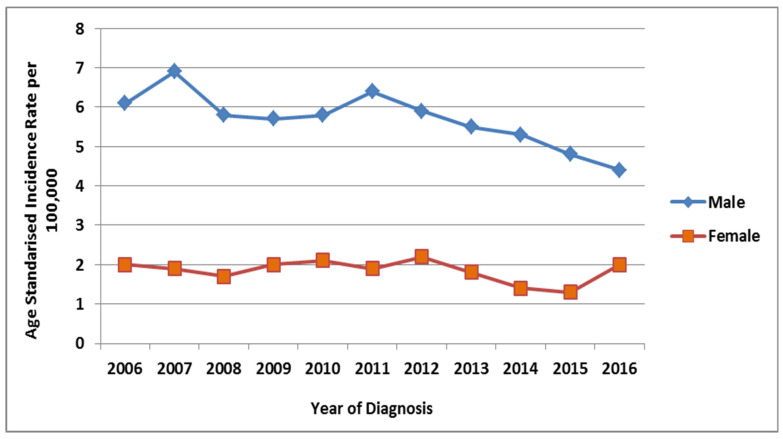
Age-standardized incidence rate of lung cancer per 100,000 in Saudi males and females.

The ASIR of lung cancer stratified according to the year of diagnosis per 100,000 males from 2006 to 2016 was calculated. An increase was observed from 2006 to 2007, it decreased from 2007 to 2008, remained stable until 2010, then increased in 2011 and decreased in 2012, then remained stable until 2014 and decreased from 2014 to 2016 (Figure 6). The ASIR of 6.9 per 100,000 males was the highest rate recorded in 2007, while it was lowest at 4.4 per 100,000 males in the year 2016. The overall ASIR of lung cancer from 2006 to 2016 per 100,000 males was 5.7 (95% CI, 5.2–6.2). Additionally, the overall age-standardized incidence sex ratio was 3.2. An independent sample *t*-test was also applied on ASIR between males and females and found that ASIRs were significantly higher in males in comparison to females (t(20) is 16.988, *p* < 0.0001).

The ASIR of lung cancer cases was stratified according to the year of diagnosis in Saudi Arabia, from 2006 to 2016 from the SCR among females (Figure 6). We observed a slight decrease from 2006 to 2008, then it increased in 2009, remained stable until 2013, then decreased from 2013 to 2015 and increased in 2016. The highest ASIR was recorded in the year 2012 at 2.2 and the lowest at 1.3 per 100,000 females in 2015. The overall ASIR from 2006 to 2016 was 1.8 per 100,000 females (95% CI, 1.6–2).

**Figure 7:**
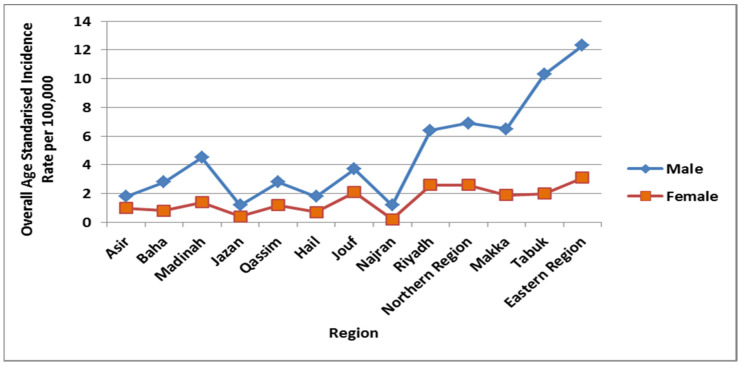
A: Overall ASIR of lung cancer cases per 100,000 among males and females according to administrative regions during 2006 to 2016.

We also identified the overall ASIR of lung cancer from 2006 to 2016 categorized by the different regions of Saudi Arabia per 100,000 males. The Eastern region had the highest overall ASIR at 12.3, followed by the Tabuk region at 10.3 per 100,000 males (Figure 7). It was found that the Kruskal–Wallis analysis was more statistically significant for these regions than other areas of Saudi Arabia (*p* < 0.001). The lowest overall ASIRs were found in the Jazan and Najran region at 1.2 and Hail and Asir at 1.8 per 100,000 males. Moreover, the highest age-standardized incidence sex ratio per 100,000 males was observed in Najran at 6, followed by Tabuk at 5.2 and the Eastern region at 4 (Figure 7).

We also identified the overall ASIR among females categorized according to different region of Saudi Arabia from 2006 to 2016 (Figure 7). The Eastern region was reported with the highest overall ASIR at 3.1, followed by Riyadh and the Northern region at 2.6 per 100,000 females. It was observed that the Kruskal–Wallis test was more statistically significant for these regions in comparison to other areas of Saudi Arabia (*p* < 0.001). The lowest overall ASIR was reported in the Najran region at 0.2, followed by Jazan at 0.4 per 100,000 females (Figure 7).

To analyze the pattern of lung cancer in different administrative regions of Saudi Arabia, differences in CIRs and ASIRs between 2006 and 2016 were identified (Table 2). The highest changes in rates were documented in the Jouf region among males (CIR: 3.3, ASIR: 5). The highest changes in rates were documented in Tabuk region among females (CIR: 3, ASIR: 1.7).

## 7. Lung Cancer: Stage Wise Distribution among Males and Females

**Figure 8:**
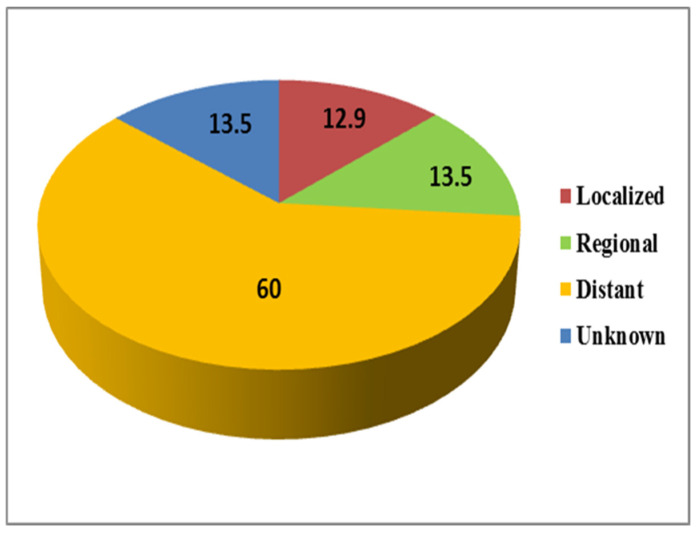
Overall percentage stage distribution of lung cancer in males during 2006 to 2016.

The overall percentage of stage distribution of lung cancer from 2006 to 2016 was also investigated among males. The most frequently diagnosed cases of lung cancer were observed at the distant stage (60%), followed by the regional and unknown stage (13.5%) and localized stage (12.9%) (Figure 8).

**Figure 9:**
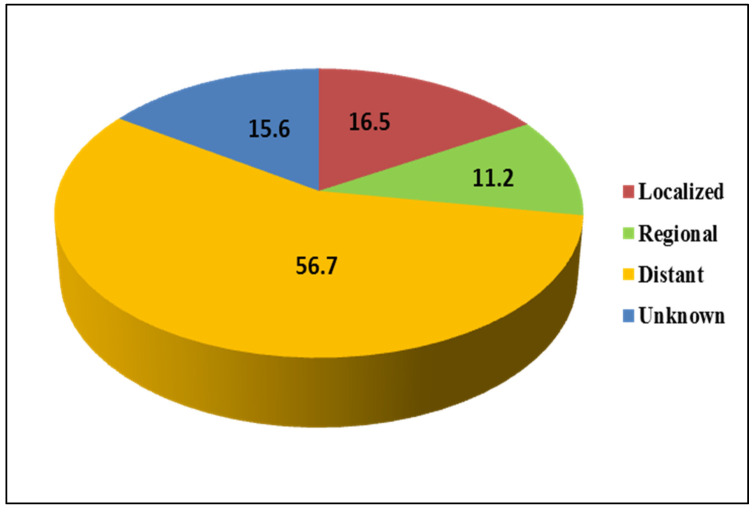
Overall percentage stage distribution of lung cancer in females during 2006 to 2016.

The overall percentage of stage distribution of lung cancer from 2006 to 2016 among females was also investigated (Figure 8). Lung cancer was most often diagnosed at the distant stage (56.7%), followed by the localized (16.5%) and unknown stages (15.6%). The lowest percentage of lung cancer was diagnosed at the regional stage (11.2) (Figure 9).

## 8. Discussion

According to GLOBOCAN 2018 estimates, lung cancer is the leading cause of mortality in males among most nations in eastern Europe, particular nations in eastern Asia (China) and southeastern Asia (Myanmar, the Philippines, and Indonesia), western Asia (particularly in the former Soviet Union) and northern Africa. The highest incidence rates were reported in Micronesia/Polynesia, in much of Europe, notably in eastern Europe (Hungary had an ASR of 77.4 per 100,000 males) and in eastern Asia (in China, Japan, and the Republic of Korea, rates were above 40 per 100,000 males). In women, lung cancer is the leading cause of cancer mortality among twenty-eight nations. The highest incidence rates were documented in North America, Northern and Western Europe (especially Denmark and the Netherlands), and Australia/New Zealand, with Hungary first on the list [2].

The age-standardized incidence rate (ASIR) and crude incidence rate (CIR) of lung cancer are crucial to be investigated and updated in different regions of Saudi Arabia. The aim of the current study is to identify the CIR and ASIR pattern from 2006 to 2016 among lung cancer cases in Saudi Arabia. It is the first descriptive epidemiological study that evaluated the spatial/temporal distribution of lung cancer among males and females from 2006 to 2016 in different regions of Saudi Arabia that is based on the PubMed database. In this present study, the actual state of lung cancer trend was examined and the significance of lung cancer in Saudi Arabia population was explored.

In this study, the highest overall ASIRs for lung cancer from 2006 to 2016 among males were documented in the Eastern and Tabuk regions. Males of these regions are greatly exposed to lung-cancer-associated risk factors as compared to other regions, which can be the most probable explanation. Additionally, an increase in ASIR among males in Eastern and Tabuk region can also be related to other factors such as genetics, lifestyle habits, and environmental risk factors. Hence, to better understand the key risk factors linked to the rise of ASIR for lung cancer in the Eastern and Tabuk region, there is a necessity of detailed epidemiological analysis. Another likely justification for the high incidence rate in these regions may be the existence of industries [12], an advanced healthcare facility and its accessibility, and Vitamin D deficiency [13]. Two studies demonstrated that increased concentration of circulating vitamin D may decrease lung cancer risk [14,15]. A study suggested the deficiency of vitamin D in 83.6% of the Arabian population [16]. In Saudi University students, 100% severe Vitamin D deficiency was observed [17]. Another study also demonstrated that the Eastern region had a high incidence of lung cancer, most likely due to the presence of chemical and petroleum industries [18]. The lowest overall ASIR for lung cancer from 2006 to 2016 among males was recorded in the Jazan and Najran region. The most probable reason could be the presence of more particular protective factors in the Jazan and Najran region than for males of higher ASIR regions. Other possible explanations may include a lesser healthcare facility and less accessibility, lesser industrialization, less Westernization, and less screening in comparison to regions with higher incidence.

Many studies showed that intake of vegetables and fruits reduced the risk of lung cancer among current smokers, mainly the cruciferous vegetables [19,20]. Dietary and supplemental calcium consumption were found to be in inverse relationship with lung cancer risk among female nonsmokers and current smokers, especially for lung adenocarcinoma [21,22]. Moreover, the risk of lung cancer was inversely linked with consumption of total iron in females, whereas total magnesium consumption elevated the risk among males and current smokers, and there was no significant relationship of lung cancer risk with selenium, copper, and zinc. Another study from Europe suggested that lower lung cancer risk was linked to higher levels of serum vitamin B and methionine among current, former, and never smokers [23]. Many findings suggested that increased risk of lung cancer mortality was associated with diabetes [24,25], and an independent predictor of lung cancer survival was baseline fasting plasma glucose level [26]. Type 2 diabetes burden is gradually increasing in Saudi Arabia because of urbanization, physical inactivity, population growth, and intake of unhealthy diet [27,28,29]. Diabetes prevalence was found to be lowest in the Jazan region [30], and we also observed the lowest ASIR in the Jazan region. Investigation of these factors regarding lung cancer may provide better understanding of this disease and may be helpful in reducing its burden.

There are several problems that should be tackled to achieve optimal lung cancer management in Saudi Arabia. Firstly, there is an absence of a systematic approach for avoiding lung cancer in the primary care setting. Mostly, lung cancer is diagnosed in advanced, difficult to manage stages. Worldwide, early-stage-diagnosed cases make up only 15% of cases, and Saudi Arabia is within the international range, with only 14% of cases diagnosed early with localized tumors [31]. Awareness of the primary and public health care providers about screening, smoking, and early detection is needed to attain the higher numbers. Though national efforts are there in smoking cessation initiatives, smoking remains widespread, prominently through hookah or shisha (water pipe) use in recent years. Consumption of tobacco products and smoking has been considered one of the significant reasons for lung cancer worldwide. Saudi Arabia being the signatory member state for the WHO framework convention on Tobacco Control (FCTC) since 2003, an anti-smoking, anti-tobacco use program has been imitated to control the tobacco epidemic in the country. One finding suggested that during the past year, only 53.2% of ever-smokers have reported obtaining assistance on quitting smoking from health care professionals [32,33]. However, high numbers of smokers in youth and secondhand smoke exposure are still there. Smoking is the major burden on adult’s health and the economy of Saudi Arabia [34]. Therefore, smoking cessation campaigns and awareness programs are required to be conducted on the national level, mainly for adults and university students, to provide them with information about smoking risks regarding lung cancer. Upcoming studies should give emphasis to assessing the knowledge level and attitude of Saudi Arabia’s general population towards smoking risk as main causative factor for lung cancer [35,36]. Secondly, major cities have all the tertiary cancer facilities, and individuals living in remote areas find difficulty in accessing cancer care facilities. The Ministry of Health is functioning on various parallel schemes for providing cancer facilities in small cities.

Despite the lack of time distribution and geographic data regarding lung cancer, we intended to study the trends and patterns of lung cancer between 2006 to 2016. Findings of this descriptive epidemiological study would be helpful in formulating better public health policy for lung cancer in Saudi Arabia and may be valuable in making a future hypothesis of possible lung cancer risk factors in the most frequently affected region by prospective epidemiological studies that identify the association of disease and exposure.

## 9. Conclusions

The present study concludes a rise in crude incidence rates and age-standardized incidence rates for lung cancer among the Saudi population. Among males, the Eastern region reported the highest overall ASIR, followed by the Tabuk region, and among females, the highest overall ASIR was reported in the Eastern region, followed by Riyadh and the Northern region. The incidence of lung cancer was reported highest in the age group of more than 75 years. The study will aid in generating lung-cancer-related epidemiological information about the Saudi Arabian population and will ultimately help in understanding cancer trends. Furthermore, this study will help to manage the incidence and mortality of cancer in the future and be implemented in screening and cancer prevention strategy in Saudi Arabia.

## Figures and Tables

**Table 1:** Number and percentage of lung cancer in males and females during 2006 to 2016.

Year	Total Lung Cancer Incidences (All Ages in Males)	%	Total Incidences of Lung Cancer (All Ages in Females)	%
2006	232	6.8	80	7
2007	323	9.5	89	7.8
2008	285	8.4	82	7.2
2009	276	8.1	103	9
2010	292	8.6	105	9.2
2011	343	10.1	109	9.5
2012	336	9.9	127	11.1
2013	326	9.6	109	9.5
2014	354	10.4	98	8.6
2015	324	9.6	92	8.1
2016	297	8.8	148	13
Total	3388	100	1142	100
Overall	308	9.1	104	9.1
%	100		100	

**Table 2:** The difference in CIR and ASIR between 2006 and 2016.

Region	Sex	CIR/ASIR	2006	2016	Difference
Asir	Male	CIR	1.2	0.9	−0.3
		ASIR	1.8	1.1	−0.7
	Female	CIR	0.4	1.3	0.9
		ASIR	0.7	1.4	0.7
Baha	Male	CIR	2.5	2.2	−0.3
		ASIR	2.9	3.2	0.3
	Female	CIR	0	0.5	0.5
		ASIR	0	0.6	0.6
Madinah	Male	CIR	2.7	2.1	−0.6
		ASIR	4.9	3.3	−1.6
	Female	CIR	1.3	0.4	−0.9
		ASIR	2.4	0.5	−1.9
Jazan	Male	CIR	1	0.5	−0.5
		ASIR	1.4	0.8	−0.6
	Female	CIR	0	0.3	0.3
		ASIR	0	0.4	0.4
Qassim	Male	CIR	1.2	1.6	0.4
		ASIR	2	2.7	0.7
	Female	CIR	0.9	0.6	−0.3
		ASIR	1.3	1	−0.3
Hail	Male	CIR	0	0.4	0.4
		ASIR	0	0.5	0.5
	Female	CIR	0	0.7	0.7
		ASIR	0	1	1
Jouf	Male	CIR	2.5	5.8	3.3
		ASIR	5.1	10.1	5
	Female	CIR	1.2	1.6	0.4
		ASIR	3.5	2.3	−1.2
Najran	Male	CIR	1.1	0.5	−0.6
		ASIR	2.2	0.6	−1.6
	Female	CIR	0	0	0
		ASIR	0	0	0
Riyadh	Male	CIR	2.7	2.9	0.2
		ASIR	6.3	4.6	−1.7
	Female	CIR	1.2	2.2	1
		ASIR	2.5	3.4	0.9
Northern Region	Male	CIR	1.6	2.1	0.5
		ASIR	5.5	4.2	−1.3
	Female	CIR	1.6	2.1	0.5
		ASIR	4.4	4	−0.4
Makka	Male	CIR	3.7	3.4	−0.3
		ASIR	6.6	4.5	−2.1
	Female	CIR	1.2	1.2	0
		ASIR	2	1.4	−0.6
Tabuk	Male	CIR	2.8	3.8	1
		ASIR	7.9	8	0.1
	Female	CIR	0.3	2	1.7
		ASIR	1.1	4.1	3
Eastern Province	Male	CIR	3.7	5.1	1.4
		ASIR	10.4	9.6	−0.8
	Female	CIR	1.1	2.5	1.4
		ASIR	2.4	3.7	1.3

Abbreviations: CIR (crude incidence rate), ASIR (age-standardized incidence rate).

## Data Availability

The data used to support the findings of this study are included within the article.

## Abbreviations

CIR	Crude incidence rate
AIR	Age-specific incidence rate
ASIR	Age-standardized incidence rate
